# The Emerging Role of Epitranscriptomics in Cancer: Focus on Urological Tumors

**DOI:** 10.3390/genes9110552

**Published:** 2018-11-13

**Authors:** João Lobo, Daniela Barros-Silva, Rui Henrique, Carmen Jerónimo

**Affiliations:** 1 Cancer Biology and Epigenetics Group, Research Center of Portuguese Oncology Institute of Porto (GEBC CI-IPOP), R. Dr. António Bernardino de Almeida, 4200-072 Porto, Portugal; joaomachadolobo@gmail.com (J.L.); daniela.barros.silva94@gmail.com (D.B.-S.); henrique@ipoporto.min-saude.pt (R.H.); 2 Department of Pathology, Portuguese Oncology Institute of Porto (IPOP), R. Dr. António Bernardino de Almeida, 4200-072 Porto, Portugal; 3 Department of Pathology and Molecular Immunology, Institute of Biomedical Sciences Abel Salazar, University of Porto (ICBAS-UP), Rua Jorge Viterbo Ferreira 228, 4050-513 Porto, Portugal

**Keywords:** bladder cancer, epitranscriptomics, eraser, kidney cancer, m^6^A, prostate cancer, reader, RNA modifications, testicular cancer, writer

## Abstract

Epitranscriptomics has gained ground in recent years, especially after the advent of techniques for accurately studying these mechanisms. Among all modifications occurring in RNA molecules, N6-methyladenosine (m^6^A) is the most frequent, especially among mRNAs. m^6^A has been demonstrated to play important roles in many physiological processes and several disease states, including various cancer models (from solid to liquid tumors). Tumor cells’ epitranscriptome is indeed disrupted in a way to promote cancer-prone features, by means of up/downregulating m^6^A-related players: the so-called writers, readers and erasers. These proteins modulate m^6^A establishment, removal and determine mRNAs fate, acting in a context-dependent manner, so that a single player may act as an oncogenic signal in one tumor model (methyltransferase like 3 (METTL3) in lung cancer) and as a tumor suppressor in another context (METTL3 in glioblastoma). Despite recent advances, however, little attention has been directed towards urological cancer. By means of a thorough analysis of the publicly available TCGA (The Cancer Genome Atlas) database, we disclosed the most relevant players in four major urogenital neoplasms—kidney, bladder, prostate and testicular cancer—for prognostic, subtype discrimination and survival purposes. In all tumor models assessed, the most promising player was shown to be Vir like m^6^A methyltransferase associated (VIRMA), which could constitute a potential target for personalized therapies.

## 1. RNA Modifications in Brief: From Epigenetics to Epitranscriptomics

In the past few years, RNA modifications have caught the scientific community’s attention. Expanding the scope of epigenetics, which comprises a group of chromatin-based mechanisms including chemical and conformational modifications of DNA and/or histones [1], “epitranscriptomics” (also called “RNA Epigenetics” [2]) relates to modifications in RNA molecules, and has emerged as a pivotal player in several biologic and disease processes [3].

More than 140 RNA modifications have been discovered so far [4,5,6] including, for instance, methylation (N7-methylguanosine [m^7^G], N6-methyl-2′-*O*-methyladenosine [m^6^Am], 2′-*O*-methylation [Nm], N6-methyladenosine [m^6^A], N1-methyladenosine [m^1^A], 5-methylcytosine [m^5^C] and 5-hydroxymethylcytosine [hm^5^C]), RNA editing (adenosine-to-inosine (A-to-I), pseudo-uridine (ψ), among others [7,8]). New modifications are emerging every day, such as KDka427 (a modification with a thioacetal structure [4]). They have been reported in various types of RNAs, including messenger (mRNAs) and noncoding (ncRNAs), such as transfer (tRNAs), ribosomal (rRNAs), small nuclear (snRNAs) and long noncoding (lncRNAs) [5]. Importantly, contrarily to DNA modifications that primarily regulate gene transcription, RNA modifications regulate the many aspects of RNAs fate, including localization, splicing, nuclear export, targeting for destruction, stability, secondary structure and efficiency of translation, ultimately allowing the formation of a functional RNA molecule. They accomplish this in a context-dependent manner, being site-specific and RNA-species-specific (i.e., the same modification can have opposing effects depending on the context it occurs in) [9].

**Figure 1:**
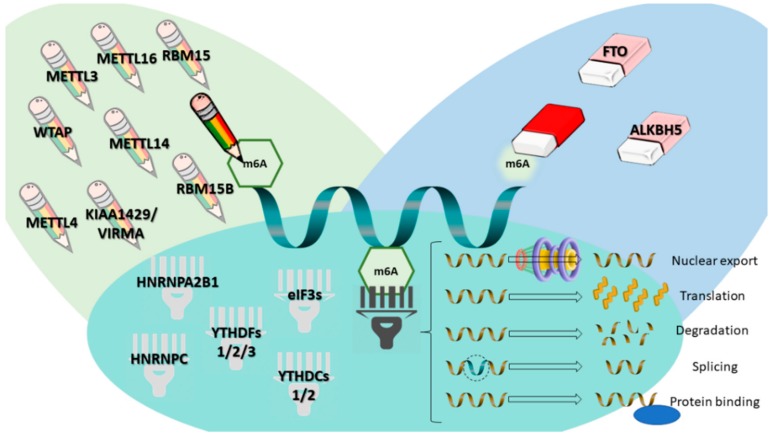
m^6^A modification and m^6^A-related proteins (writers, erasers and readers), and their respective functions. Writers are illustrated as “pencils”, erasers as “pencil erasers” and readers as “barcode readers.” METTL4, 14, 3, 16—methyltransferase like 4, 14, 3 or 16; KIAA1429/VIRMA—Vir like m^6^A methyltransferase associated; RBM15 or 15B—RNA binding motif protein 15 or 15B; FTO—fat mass and obesity associated; ALKBH5—α-ketoglutarate dependent dioxygenase 5; WTAP—Wilms’ tumor 1-associating protein; YTHDF 1, 2 and 3—YTH domain family proteins 1, 2 and 3; YTHDC 1 and 2—YTH domain-containing proteins 1 and 2; eIF3—eukaryotic initiation factor 3; HNRNPC—heterogeneous nuclear ribonucleoprotein C; HNRNPA2B1—heterogeneous nuclear ribonucleoprotein A2-B1; m^6^A—N6-methyladenosine.

Among RNA modifications, m^6^A (first reported in 1974 [10], but not given full acceptance until the advent of methodologies for mapping its location) is the most abundant in eukaryotic mRNAs and lncRNAs (m^6^A/A = 0.1–0.6%) [11], and will be the focus of this review. It is not randomly distributed across transcripts, being particularly enriched at 3′ untranslated regions (3′UTRs), around stop codons and within internal long exons [7,12,13,14]. The finding of this non-random post-transcriptional mRNA methylation pattern (the mRNA “epitranscriptome”), along with the discovery of adenosine methyltransferases (“writers”), m^6^A demethylating enzymes (“erasers”) and m^6^A binding proteins (“readers”), indicate that mRNAs undergo methylation as a fine-tuning mechanism which reversibly and dynamically regulates their overall activity (similar to methylation of DNA molecules) [15,16].

m^6^A has been shown to play important roles in regulating gene expression and phenotypes in both health and disease. In this line, great effort has been made to find and improve methodologies for detecting and profiling these alterations (with transcriptome-wide analysis being considered “Method of the Year” by Nature Methods [17]), and new methodologies with different approaches are being uncovered every day [11,18,19]. m^6^A is, indeed, the most prevalent internal modification of mRNAs. Its respective writer (methyltransferase like 3 (METTL3), which writes the methyl code onto RNA) along with other components of the methylation complex (METTL14, METTL4, METTL16, Wilms Tumor 1-Associating Protein (WTAP), Vir like m^6^A methyltransferase associated (KIAA1429/VIRMA), RNA binding motif protein 15 (RBM15), RBM15B), its erasers (proteins that remove the methyl code from RNA, such as fat mass and obesity related (FTO) and α-ketoglutarate dependent dioxygenase 5 (ALKBH5)) and its readers (proteins that recognize m^6^A, decode it and transform it into a functional signal, targeting RNAs for their final destination and initiating downstream processes, such as YTH domain family proteins (YTHDF) 1, 2 and 3, YTH domain-containing proteins (YTHDC) 1 and 2, eukaryotic initiation factor 3 (eIF3), heterogeneous nuclear ribonucleoprotein C (HNRNPC) and heterogeneous nuclear ribonucleoprotein A2-B1 (HNRNPA2B1)) have been already identified (Figure 1); these facts, in parallel with the improvement of methodology for accurately profiling m^6^A, have increased the interest in studying the impact of modifying m^6^A levels by changing the expression of these proteins in various disease states [8,14,20,21,22,23,24].

In this vein, m^6^A has been explored in many perspectives and different contexts in the past few years, having been shown to play important roles in very diverse biological mechanisms and related diseases, including metabolism/obesity, circadian rhythm, immune response, viral replication, gametogenesis/infertility, embryogenesis/stem cell differentiation, neurologic development/deficits, and also in cancer [3,16,25,26,27,28,29,30,31,32,33,34,35,36,37,38,39].

## 2. m^6^A Modification in Non-Urological Malignancies: Literature Review

The epitranscriptome of cancer cells has been demonstrated to be disrupted [40], and associations with dysregulation of expression of m^6^A-related proteins (i.e., their writer, readers and erasers) have been increasingly found in many neoplasms [41]. It is reasonable to think that, by modifying the epitranscriptome, tumor cells modify the fate of many target transcripts, which might influence many aspects of cancer progression, including growth and proliferation, invasiveness, migration and metastatic spread, stemness maintenance and differentiation, response to immune surveillance and to stress, among others [21]. Again, and in accordance with the context-dependent role of RNA modifications, evidence has shown that both writers and erasers can assume an oncogenic or tumor suppressor role in different tumor models (for instance, the writer METTL3 may act as oncogene in lung adenocarcinoma and as tumor suppressor in glioblastoma) [21,42].

Modifications in m^6^A levels and/or m^6^A-related proteins expression have been found in a broad spectrum of cancer types. Thus, targeting m^6^A regulatory mechanisms might constitute a new form of cancer treatment [43], especially for suppressing cancer stem cells [42,44]. Clinical trials with drugs targeting oncogenic regulators of the epitranscriptome (such as FTO inhibitors like Citrate and R-2-hydroxyglutarate (R-2HG)) are needed and expected for the near future [37,45].

Polymorphisms in intron 1 of FTO have been associated with a higher risk for development of many neoplasms; however, a metanalysis concluded that, except for pancreatic cancer, the risk was mainly due to body mass index (BMI) [46,47]. However, a single-nucleotide polymorphism (SNP) in FTO intron 8 was found to be associated with a higher risk for melanoma [48], and, as for breast cancer (BCa), another SNP in FTO intron 1 was identified as a susceptibility locus for estrogen-negative BCa [49], both not explained by BMI. FTO was also overexpressed in BCa (particularly in human epidermal growth factor receptor 2 (HER2)-positive tumors) and was also shown to play a role in triple-negative, pan-resistant, inflammatory breast cancer cell lines [50,51]. Also in BCa, a link between hypoxia, tumor invasiveness/metastasis and m^6^A has been proposed, with hypoxia-inducible factors (HIFs) leading to increased mRNA expression of the pluripotency factor homeobox transcription factor Nanog (NANOG) (and subsequent BCa stem cells specification) by means of m^6^A demethylation by the eraser ALKBH5 [52,53]. Furthermore, a positive feedback loop involving HBXIP/miR let-7g/METTL3 was reported to promote BCa progression and proliferation [54].

The writer METTL3 was also shown to be upregulated in various solid tumors, including hepatocellular carcinoma, associated with poor prognosis. In this tumor model, METTL3-mediated m^6^A modification targets suppressor of cytokine signaling 2 (SOCS2), promoting its degradation, in a process dependent of YTHDF2 reader [55]. More recently, YTHDF1 proved also to be upregulated in hepatocellular carcinoma, associated with more advanced stages and poorer survival, contrarily to METTL14, which promotes metastatic potential when downregulated [56,57]. In addition, m^6^A and related proteins are implicated in treatment resistance, as shown in pancreatic cancer cells, in which knockdown of the writer METTL3 improved sensitivity to both chemo- and radiation therapy [58], clearly demonstrating the rationale for using treatments targeting m^6^A modulators. Finally, the reader YTHDF2 was shown to display both diagnostic and prognostic value in pancreatic cancer and to regulate the epithelial-to-mesenchymal transition (EMT) phenomenon [59], whereas WTAP was found to promote migration and invasion in cholangiocarcinoma [60].

Concerning colorectal cancer, the reader YTHDF1 seems to be of paramount importance in disease progression, with immunoexpression associating with unfavorable prognosis disease parameters and poorer survival. Again, the knockdown of YTHDF3 sensitized cancer cells to chemotherapy and, additionally, oncogene c-Myc was found to drive YTHDF1 expression [61]. Moreover, and besides the several RNA editing modifications reported, increased expression of the reader YTHDC2 in colorectal cancer promotes metastatic spread by upregulating hypoxia inducible factor 1 subunit α (HIF-1α) [62,63]. FTO overexpression was shown to impact on prognosis in gastric cancer patients, associated with poor differentiation, lymph node metastases, tumor stage and poor survival [64].

In cervical cancer, a lower amount of m^6^A mRNA modification was associated with disease progression and poor prognosis (higher International Federation of Gynecology and Obstetrics (FIGO) stage, recurrence, metastases and survival), and further manipulation of m^6^A levels in cell lines by altering the expression of respective writers and erasers resulted in increasing or decreasing disease aggressiveness, respectively [65]. More recently, it was demonstrated that the eraser FTO is also upregulated in cervical cancer and leads to chemo- and radiation therapy resistance by demethylating the mRNA transcripts of its target, β-catenin [66]. In addition, a recent study in endometrial cancer has elegantly shown that decreased m^6^A caused by a mutation in METTL14 or downregulation of METTL3 ultimately leads to increased proliferation by activating the AKT signaling pathway [67].

m^6^A modification in mRNA of glioblastoma stem cells regulates their capacity of self-renewal and tumorigenesis, with overexpression of writers (METTL3 and METTL14) and downregulation of erasers (FTO and ALKBH5) inhibiting tumor growth. In addition, high levels of the eraser ALKBH5 associated with poor prognostic features and METTL3 associated with radiation therapy resistance [68,69,70]. This finding may be explored as a potential therapeutic target. Moreover, in lung cancer, another aggressive neoplasm, METTL3 was shown to act as an oncogene, inducing tumor growth and proliferation, also promoting translation of important genes such as epidermal growth factor receptor (EGFR) and tafazzin (TAZ) [71]. An interaction between molecules like microRNAs and m^6^A alterations was also depicted, with miR-33a inhibiting lung cancer cells proliferation by targeting METTL3 [72].

m^6^A has been demonstrated to have an impact in biogenesis of hematolymphoid neoplasms, as well. It was shown that mutations in m^6^A-related proteins confer poor prognosis in acute myeloid leukemia (AML) [73]. Mutations in writers (METTL3, METTL14, WTAP, RBM15) promote and maintain leukemogenesis in AML [74,75,76,77,78,79], whereas overexpression of the eraser FTO in AML cell lines also promoted proliferation and decreased apoptosis [80]. Moreover, FTO plays a role in response to all-*trans*-retinoic acid (ATRA) and, interestingly, D-2-hydroxyglutarate (D2-HG) (the metabolite accumulated in isocitrate dehydrogenase 1 and 2 (IDH1/2)-mutant leukemias (20% of AMLs)) functions as an inhibitor of FTO demethylase, meaning that FTO expression is context-dependent and has to be interpreted according to IDH mutational status [81,82]. A summary of the findings presented in this section is depicted in Table 1.

## 3. m^6^A Modifications in Urological Tumors: Analysis of The Cancer Genome Atlas Database

Although RNA modifications have been analyzed in several tumor models in recent years, little attention has been paid to urological cancer. One of our main research goals is to uncover and characterize new epigenetic modifiers in urological malignancies, to be applied in diagnosis, prognosis and disease monitoring. In this line, we performed an in silico analysis of the publicly available The Cancer Genome Atlas (TCGA) database regarding m^6^A-related proteins (writers, erasers and readers) in the four main urological cancers: bladder (BlCa), kidney (KCa), prostate (PCa) and testicular cancer. For that purpose, the online resource cBioPortal for Cancer Genomics [83] was used, with the user-defined entry gene set “METTL3, METTL14, METTL4, METTL16, WTAP, VIRMA, RBM15, RBM15B, FTO, ALKBH5, YTHDF1, YTHDF2, YTHDF3, YTHDC1, YTHDC2, EIF3A, HNRNPC and HNRNPA2B1”. Statistical analysis with the available data was performed with Microsoft Excel 2016, (Microsoft, Redmond, Washington, USA), GraphPad Prism 6 (Prism, San Diego, California, USA) and IBM SPSS Statistics v.24 (Armonk, NY, USA). Distribution of continuous variables between groups was compared using the nonparametric Mann–Whitney test. Correlations between continuous variables were assessed with Spearman’s non-parametric correlation test. Co-occurrence/mutual exclusivity of alterations in pairs of genes was estimated with odds ratio (OR). Biomarker performance was assessed through receiver operating characteristics (ROC) curve construction. In brief, for each transcript, an ROC curve was constructed plotting sensitivity (true positive) against 1-specificity (false positive). A cut-off was established by ROC curve analysis (sensitivity + (1-specificity)), to maximize both sensitivity and specificity. In addition, area under the curve (AUC) and biomarker performance parameters, including sensitivity, specificity, positive predictive value (PPV), negative predictive value (NPV) and accuracy, were ascertained. Survival curves were plotted using the Kaplan–Meier method and log rank test was used for survival analysis. A *p*-value equal or inferior to 0.05 was considered statistically significant.

### 3.1. Prostate Cancer

PCa is a major public health concern in male gender mainly due to the growth and aging of the global population [84]. It is a highly prevalent malignancy, being the second most common cancer and the fifth leading cause of death from cancer in men, mostly due to aggressive and metastatic disease [85]. This neoplasia is usually clinically silent until extra-prostatic invasion or metastization occur, being a complex and heterogeneous disease, ranging from clinically indolent to highly aggressive [86,87]. At its earliest stages, PCa is sensitive to androgen-deprivation therapy, which is the mainstay treatment for advanced disease. Nevertheless, patients eventually develop castration-resistance and progress to lethal PCa [88].

Concerning patient management, clinicians face three major challenges: to distinguish PCa from benign prostatic hyperplasia and other cancer mimickers; to discriminate indolent from aggressive disease; and to foresee patients that will undergo disease progression and develop metastatic disease [89]. Epigenetic alterations are a common trait in PCa and are involved in disease onset and progression. Despite their exact roles are still not fully understood, the fact that they occur at a higher rate and in an earlier point than mutations makes them very attractive biomarkers for diagnosis, prognosis and follow-up purposes [90].

The TCGA database for PCa includes 499 samples from 498 patients, with a median age at diagnosis of 61 years. Patients were American Joint Committee on Cancer (AJCC) stages II, III and IV in 187/490 (38.2%), 293/490 (59.8%) and 10/490 (2.0%) cases. Regarding Gleason score and respective grade groups (GG), patients were classified from GG 1 to 5 (GG1 = 8.8%, GG2 = 29.4%, GG3 = 20.5%, GG4 = 12.9% and GG5 = 28.4%). Ten patients died and 58 experienced disease recurrence/progression, resulting in an overall survival (OS) and disease-free survival (DFS) at 10 years of 68% and 53%, respectively.

Overall, the 18-gene list was found to be altered in 307/499 samples (61.5%), mainly by mRNA upregulation (*n* = 111, 22.2%). mRNA downregulation also occurred in 54 cases (10.8%), and multiple alterations were depicted in 88 cases (17.6%). Individual mutations, amplifications, deep deletions, protein upregulations or protein downregulations were seldomly observed (*n* = 7, 1.4%; *n* = 7, 1.4%; *n* = 34, 6.8%; *n* = 2, 0.4% and *n* = 4, 0.8%, respectively).

Deregulation of VIRMA, a component of the methylation complex, and of the readers YTHDF3 and YTHDC2 are of particular interest, constituting the most commonly altered genes in the pathway (18%, 13% and 11% of the samples, respectively). The remainder genes analyzed depicted alterations in less than 10% of samples. In addition, no mutations are described for YTHDF3 and YTHDC2 that may explain the deregulation, and only three samples disclosed a missense mutation in VIRMA. There was also a modest correlation between VIRMA and YTHDF3 mRNA expression in PCa samples (correlation coefficient: 0.62).

Analysis of alterations in the various pairs of genes showed two gene pairs with significant co-occurrent alterations. The strongest associations, with Bonferroni correction, included VIRMA and YTHDF3 (log OR > 2 and *p* < 0.001).

Regarding clinicopathologic correlates, VIRMA and YTHDF3 mRNA expression levels were significantly higher in stage III/IV compared to stage II tumors (*p* ≤ 0.0001 and *p* = 0.0454, respectively). In the same line, higher VIRMA and YTHDF3 transcript levels associated with higher GG (GG2-5 vs. GG1, *p* = 0.0198 and *p* = 0.0215, respectively), again suggesting higher expression of these players in more aggressive diseases. None of the genes tested impacted on overall survival (OS) or disease-free survival (DFS).

Although still largely unexplored, there is already a study (using both cell lines and human tissues from 35 patients) reporting m^6^A alterations in PCa. Specifically, the authors report that YTHDF2, an m^6^A reader, is regulated by miR-493-3p and its upregulation is involved in the m^6^A modification and malignant progression [91].

### 3.2. Testicular Cancer

Testicular germ cell tumors (TGCTs) comprise more than 95% of all testicular neoplasms, and are grouped into two major families according to the most recent World Health Organization (WHO) classification: the germ-cell neoplasia in situ (GCNIS)-related tumors (the most frequent, which include seminomas (SEs) and non-seminomatous Tumors (NSTs), two subgroups with very distinct behavior and clinical impact), and the GCNIS-unrelated ones [92].

Despite representing only 1% of male cancer worldwide, they constitute the most common cancer afflicting Caucasian men between 15–44 years old, with Western lifestyle contributing to a rising incidence worldwide. They also exhibit outstanding cure rates and a dropping mortality trend in response to multimodal treatments. However, many issues are left unresolved and deserve our attention, namely the substantial proportion of patients with disseminated disease that relapse with poor prognosis, the emergence of cisplatin resistance and the considerable morbidity induced by chemo- and radiotherapy in such young patients with long survival expectancy [85,93,94,95].

Testicular germ cell tumors are remarkably heterogeneous (reflecting the complexity of this tumor model) [96] but mainly share a unifying cytogenetic background. In this line, it is only natural that various Epi-phenomena might play a fundamental role in these neoplasms. Therefore, the study of new Epi-markers might aid in tumor subtype discrimination, prognosis assessment and disease monitoring, as no accurate validated biomarkers exist for these purposes. In addition, the manipulation of these Epi-markers might provide ways of uncovering therapies with improved antitumor activity, less toxicity and that may overcome cisplatin resistance [97,98,99,100,101].

The database for TGCTs includes 156 samples from 150 patients, 65 SEs, 71 NSTs and a third category of tumors regarded as Embryonal Carcinoma (EC), composed of 20 samples. This way, the total amount of NSTs in the cohort is 91. Median age at diagnosis is 31 years. Patients were AJCC stages I, II and III in 100/126 (79.4%), 12/126 (9.5%) and 14/126 (11.1%) cases. According to the International Germ Cell Consensus Collaborative Group (IGCCCG) for metastatic disease [102], 32/43 (74.4%), 9/43 (20.9%) and 2/43 (4.7%) patients were in prognostic groups “Good”, “Intermediate” and “Poor”. Three patients died and 33 experienced disease recurrence/progression, resulting in an OS and DFS at five years of 98% and 76%, respectively.

Overall, the 18-gene list was found altered in 134/156 samples (85.9%), mainly by mRNA upregulation (*n* = 94, 70.2%). mRNA downregulation occurred in 15 cases (11.2%), and multiple alterations were depicted in 19 cases (14.2%). Like in PCa, individual mutations, amplifications, deep deletions or protein downregulations were seldomly observed (*n* = 2, 1.5%; *n* = 1, 0.7%; *n* = 2, 1.5%; and *n* = 1, 0.7%, respectively).

Paralleling our analysis on PCa, deregulation of VIRMA and the reader YTHDF3 is particularly interesting in TGCTs as well, being the two most commonly altered genes in the pathway (52% and 48% of samples, respectively). Following these two major deregulated genes, the reader HNRNPA2B1 and the writer METTL3 were also altered in 13% and 10% of the samples, respectively. The remaining genes analyzed disclosed alterations in less than 10% of samples. VIRMA and YTHDF3 are differently deregulated in SEs and NSTs (depicting alterations in 80% and 72% of SEs and in only 31% and 31% of NSTs, respectively), again mainly by mRNA upregulation. In addition, no mutations have been described for YTHDF3 that can explain its deregulation, and only one sample disclosed a missense mutation in VIRMA. There was also a strong correlation between VIRMA and YTHDF3 mRNA expression in TGCT samples (correlation coefficient 0.77).

Analysis of alterations in the various pairs of genes identified 10 gene pairs with significant co-occurrent alterations. The strongest associations included VIRMA + YTHDF3, YTHDC2 + EIF3A and METTL14 + YTHDC2 (log OR > 3 and *p* < 0.001 for all). However, the only one significant applying Bonferroni correction was precisely the VIRMA + YTHDF3 pair (log OR > 3, *p*-value < 0.001). Four gene pairs showed significant mutual exclusivity alterations, the strongest being YTHDF2 + YTHDF3 (log OR < −3, *p* = 0.002), VIRMA + HNRNPC (log OR < −3, *p* = 0.011) and YTHDF3 + HNRNPC (log OR < −3, *p* = 0.018). However, none was significant after Bonferroni correction for multiple comparisons.

Regarding subtype discrimination, mRNA expression levels of VIRMA and YTHDF3, but also the writer METTL4, the eraser ALKBH5 and the reader YTHDC1, were significantly higher in SEs compared to NSTs (*p* < 0.0001 for all). On the contrary, the writer METTL14 was significantly downregulated in SEs vs. NSTs (*p* < 0.0001). Of these genes, the best discriminative power assessed by ROC curve analysis was METTL4 (AUC = 0.91), followed by VIRMA (AUC = 0.83). Using the mRNA expression level that maximizes both sensitivity and specificity (228.04925) as cutoff, METTL4 discriminated between SEs and NSTs with 92.3% sensitivity, 82.4% specificity, 78.9% positive predictive value, and 93.8% negative predictive value, resulting in overall accuracy of 86.5%. Remarkably, METTL4 outperforms the serological markers commonly used in clinical practice (α-fetoprotein, subunit β of the human chorionic gonadotropin and lactate dehydrogenase) [95].

Furthermore, mRNA expression levels of METTL4, VIRMA and YTHDF3 were also significantly higher in stage I compared to stage II/III TGCTs (*p* = 0.0234, *p* = 0.0065 and *p* = 0.0165, respectively). Regarding survival analysis, the only genes with impact on survival were METTL4 (cases with alterations showing worse DFS, *p* = 0.0249), WTAP (cases with alterations showing worse DFS, *p* = 0.0402) and YTHDF1 (cases with alterations showing worse OS, *p* = 0.0440).

### 3.3. Kidney Cancer

Kidney cancer is the 14th most common malignancy worldwide and the 8th most prevalent cancer in Europe representing 3.5% of all adult malignancies. It is the most lethal among common urological cancers and, in 2012, there were 143,406 deaths attributable to this malignancy worldwide. Furthermore, incidence varies by gender, with men having twice the risk of women [85,103]. Due to its retroperitoneal topography, many renal masses remain asymptomatic until late stages. However, widespread use and improvement of imaging methods led to increased incidental detection of small renal masses, emphasizing the need for accurate discrimination among KCa subtypes, specifically between those which will be more aggressive and develop metastases and those that will have a more indolent growth and may be managed more conservatively [104].

According to the current World Health Organization (WHO) 2016 classification, malignant tumors are classified into three most common subtypes: clear cell renal cell carcinoma (ccRCC) (65–70%), the most common and aggressive phenotype; papillary renal cell carcinoma (pRCC) (15–20%), which has two variants, types 1 and 2; and chromophobe renal cell carcinoma (chRCC) (5–10%), the least aggressive of these [92].

The database for renal cell carcinoma includes 897 samples from 895 patients (67% male), from which 538/897 (60%) are ccRCC, 66/897 (7%) are chRCC and 293/897 (33%) are pRCC. Median patient age at diagnosis was 60 years. Patients were AJCC stages I, II, III and IV in 462/858 (54%), 102/858 (12%), 190/858 (22%) and 104/858 (12%) cases, respectively. During follow-up, 227 patients died and 189 experienced disease recurrence/progression, resulting in an OS and DFS at five years of 69% and 72%, respectively.

Overall, the 18-gene list was altered in 585/883 samples (66%). Specifically, by tumor subtype, the most commonly altered genes were: YTHDC2 (21%) and RBM15B (14%) in ccRCC, mostly due to mRNA upregulation (26.4%); VIRMA (17%) and HNRNPA2B1 (17%) in chRCC, mostly due to mRNA downregulation (26.4%); and METTL16 (19%), YTHDF1 (19%) and RBM15B (14%) in pRCC, mostly due to mRNA upregulation (26.4%).

There were two gene pairs with significant co-occurrent alterations, after Bonferroni correction, including VIRMA + YTHDF3 and RBM15B + YTHDC2 (both with log OR > 3 and *p* < 0.001). No significant gene pairs with mutually exclusive alterations were found.

VIRMA, RBM15B and YTHDC2 mRNA expression levels discriminated among ccRCC, chRCC and pRCC; transcript levels of VIRMA and YTHDC2 were lower in chRCC (*p* < 0.0001 for both) and in pRCC (*p* < 0.0001 and *p* = 0.0006, respectively) compared to ccRCCC. Contrarily, RBM15B was significantly upregulated in chRCC (*p* < 0.0001) and in pRCC (*p* < 0.0001) compared to ccRCC. Furthermore, RBM15B and YTHDC2 higher expression levels associated with advanced AJCC tumor stage (Stages II-IV vs. Stage I, *p* = 0.0361 and *p* = 0.0045, respectively). Regarding survival analysis, the only genes with impact on survival were VIRMA and YTHDC2 in ChRCC in both OS (mRNA upregulation conferring worse OS, *p* = 0.0280 and *p* = 0.0497, respectively) and DFS (mRNA upregulation conferring worse DFS, *p* = 0.0203 and *p* = 00152, respectively), and RBM15B in pRCC only in DFS (mRNA downregulation conferring worse DFS, *p* = 0.0082).

Although there is substantial lack of information regarding m^6^A alterations in KCa, Li and Tang et al. reported that higher expression of METTL3 might indicate better survival among RCC patients. Their study, which included both cell lines and human tissues from 145 patients (127 ccRCC and 18 designated as “others”), showed that this m^6^A writer might act as a tumor suppressor and may have impact on tumorigenesis and survival of KCa patients [105].

### 3.4. Bladder Cancer

Bladder cancer is the 9th most common cancer worldwide, with an estimated 430,000 new cases diagnosed in 2012. It is more prevalent in males (3/4 of all BlCa cases), representing the 6th most incident neoplasm in this group. Importantly, it is an important health issue, as recent trends follow tobacco smoking prevalence and because it was responsible for 165,000 deaths in 2012 (75% of which in men) [106,107].

The major histological type of BlCa is urothelial carcinoma. There are two major clinical, pathological and molecular forms of the disease: the non-papillary muscle-invasive tumors (with carcinoma in situ as the major precursor, being the most aggressive, more prone to progress and metastasize—25% of newly diagnosed cases) and the papillary non-muscle-invasive ones (with papillary urothelial lesions as precursors, being mainly characterized by multiple local recurrences, associated morbidity and, finally, increased risk of muscle-invasion over time—75% of newly diagnosed cases) [108].

The database for bladder urothelial carcinoma comprises muscle-invasive carcinoma only and includes 413 samples from 412 patients (73.5% male), 273/406 (67.2%) originating from the non-papillary pathway and 133/406 (32.8%) from the papillary pathway. Median patient age at diagnosis was 69 years and 387/408 (94.9%) tumors were high grade. Patients were AJCC stages I, II, III and IV in 2/409 (0.5%), 131/409 (32.0%), 141/409 (34.5%) and 135/409 (33.0%) cases. During follow-up, 181 patients died and 143 experienced disease recurrence/progression, resulting in OS and DFS at 5 years of 42% and 41%, respectively.

Overall, the 18-gene list was altered in 329/413 samples (80.0%), mainly by mRNA upregulation (*n* = 134, 40.7%) or by multiple alterations (*n* = 131, 39.8%). mRNA downregulation, mutations, amplifications, deep deletions and proteins downregulations were depicted in 20 (6.1%), 15 (4.6%), 12 (3.7%), 10 (3.0%) and 7 (2.1%) cases, respectively.

Like TGCTs, the most commonly deregulated gene was VIRMA (29% of samples), mainly by upregulation. Other frequently deregulated genes were YTHDF1 (27%), METTL4 (21%), YTHDF3 (14%) and RBM15 (13%). VIRMA was also deregulated in 33% of non-papillary tumors, but only in 20% of papillary tumors. The rate of somatic mutations in these genes was only 0.5%, 0.5% and 0.7% for YTHDF1, METTL4, and YTHDF3, respectively; VIRMA and RBM15 mutations were found in 9 (2.2%) and 12 (2.9%) cases. There was a moderate correlation between expression levels of VIRMA and YTHDF3 (correlation coefficient 0.57) and METTL14 (correlation coefficient 0.40).

There were eight gene pairs with significant co-occurrent alterations; after Bonferroni correction, the strongest ones included METTL14 + YTHDC1 (log OR 2.308, *p* = 0.042) and METTL3 + HNRNPC (log OR 2.260, *p* < 0.001). Like in TGCTs, VIRMA + YTHDF3 also tended to co-occur (log OR 1.915, *p* < 0.001). No significant gene pairs with mutual exclusive alterations were found.

High grade tumors showed significantly higher mRNA expression of VIRMA, METTL4 and YTHDF3 compared to low grade tumors (*p* = 0.003, *p* < 0.001 and *p* = 0.041, respectively), but the discriminative power was limited, the best disclosed by METTL4 (AUC 0.80). METTL4 and YTHDF1 mRNA expression levels did not, however, discriminate between papillary and non-papillary derived tumors (*p* = 0.1622 and *p* = 0.4321, respectively), but VIRMA was significantly upregulated in non-papillary tumors (*p* = 0.022), contrarily to YTHDC1 which was upregulated in papillary tumors (*p* = 0.0038). Nonetheless, the discriminative power was poor (AUC 0.59 for both).

**Figure 2:**
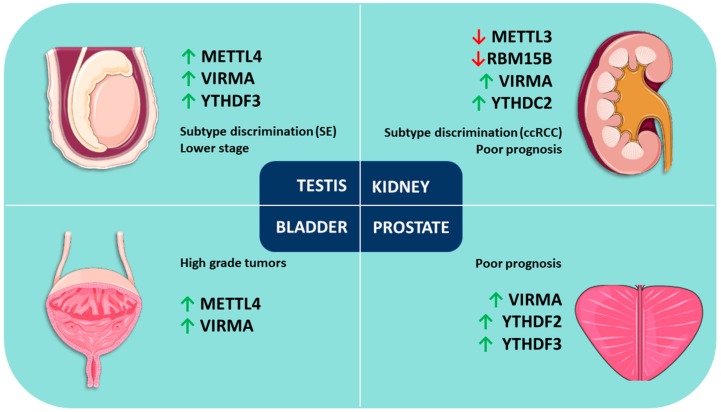
Review of m^6^A modification and related proteins in urological malignancies. Upward arrows mean “upregulation”, and downward arrows mean “downregulation.”

YTHDC1 was upregulated in stage I/II compared to stage III/IV disease (*p* = 0.0089). Regarding survival analysis, the only gene with impact on survival was WTAP (cases with alterations showing better OS, *p* = 0.0261). A summary of these findings is depicted in Table 2 and illustrated in Figure 2.

## 4. Discussion

In PCa, higher expression levels of VIRMA and YTHDF3 appear to be associated with advanced disease. The positive correlation between VIRMA and the reader YTHDF3 suggests cooperation for the establishment of m^6^A modification in these tumors. YTHDF2 expression also has an impact on disease progression as shown by Li and Meng et al. [91].

On the other hand, in TGCTs, expression levels of METTL4 and VIRMA seem promising biomarkers for discrimination between SEs and NSTs. In this tumor model, a positive strong correlation between VIRMA and the reader YTHDF3 was also observed, supporting again a cooperation between these two players for establishing m^6^A modification in urologic tumors. METTL4 expression had also an impact on DFS and associated with disease stage, as did VIRMA and YTHDF3 expression.

In KCa, VIRMA, RBM15B and YTHDC2 expression levels are auspicious biomarkers for discrimination among RCC subtypes, having impact on OS and DFS. Specifically, RBM15B and YTHDC2 associate with the advanced disease stage. Moreover, METTL3 plays a tumor suppressor role in this malignancy possibly acting as a novel marker for kidney tumorigenesis, as suggested by Li and Tang et al. [105].

Finally, in BlCa, VIRMA and METTL4 seem to be the most useful markers, as they are amongst the most commonly deregulated and they are significantly upregulated in high grade tumors. VIRMA was significantly upregulated in non-papillary tumors, but discrimination of the two major BlCa pathways using these markers remains a challenge.

Overall, regarding non-urological malignancies, upregulation of writers and/or writer-related players, tend to associate with more aggressive cancer features (poor prognosis, invasiveness, metastases and even treatment resistance). Mechanistically, this seems to imply that higher amounts of m^6^A modification in target RNAs might result in the development of cancer-prone features. Urological cancers tend to follow the same pattern, with upregulation of methylating enzymes associated with higher tumor grade and stage. The finding seems to contrast with the idea that m^6^A introduction is necessary for differentiation and that decreased m^6^A amount results in resistance to differentiation [29]. Nonetheless, exceptions exist both in urological and non-urological cancers. For instance, in TGCTs, higher VIRMA expression was found to be associated with low disease stage; in addition, in KCa, RBM15B overexpression (an eraser) associated with advanced disease at diagnosis, whereas the writer METTL3 was reported to act as a tumor suppressor. Ultimately, this might be interpreted in several ways: either the reader dictates the overall final destination of the target RNA (which can vary from degradation to increased translation), or m^6^A target RNAs may function as tumor suppressors or oncogenes. Overall, one has to take into account tumor subtype, relative expression of writers, erasers and readers, as well as the exact transcripts that are m^6^A-targeted.

Considering the ensemble of urological cancers, VIRMA upregulation stands as a common and shared trait, although in a variable proportion of cases. Considering the dissimilarity of age groups affected, as well as of risk factors, this is an intriguing observation. Nevertheless, it emphasizes the relevance of epitranscriptomics, and of m^6^A alteration in particular, in the genesis and progression of urological cancers.

## Figures and Tables

**Table 1:** Review of the literature regarding m^6^A modification and related proteins in non-urological malignancies.

Tumor Model	Methodology	Outcome	Sample Size	Author (Ref.)
**Liver**	MeRIP/RIP	METTL3 upregulation associates with poor prognosis	120 patients, cell lines and animal models	Chen M 2017 [55]
	m^6^A-Seq			
	RT-qPCR			
	WB			
	TCGA database	YTHDF1 upregulation associates with poorer stage and survival	373 patients	Zhao X 2018 [56]
	GO and KEGG enrichment analysis *			
	MeRIP/RIP	METTL14 deregulation promotes metastatic spread	130 patients and animal models	Ma JZ 2017 [57]
	RT-qPCR			
	m^6^A Dot Blot/Immunobloting			
	WB			
	IHC			
**Breast**	IHC	FTO overexpression associates with HER-2 positive Breast Cancer	79 patients	Tan A 2015 [50]
	WB	Pharmacological inhibition of FTO reduces survival of chemoresistant Inflammatory Breast Cancer cells	Cell lines	Singh B 2016 [51]
	IHC	Hypoxia induces cancer stem cell phenotype by ALKBH5-mediated m^6^A-demethylation	Cell lines	Zhang C 2016 and 2016 [52,53]
	RT-qPCR			
	MeRIP/RIP			
	WB			
	Genotyping using custom Illumina array (iCOGS)	SNP in FTP contributes to susceptibility for ER-negative cancer	6514 patients	Garcia-Closas M 2013 [49]
	MeRIP/RIP	Positive feedback loop HBXIP/miR let-7g/METTL3 promotes cancer progression	24 patients, tissue microarrays (90 breast cancer tissue samples) and cell lines	Cai X 2017 [54]
	RT-qPCR			
	m^6^A Dot Blot/Immunobloting			
	IHC and IF			
	WB			
**Melanoma**	GenoMEL *	FTO associates with higher melanoma risk	1373 patients	Iles MM 2013 [48]
**Lung**	MeRIP/RIP	METTL3 upregulation increases translation of oncogenic pathways	Cell lines	Lin and Choe 2016 [71]
	m^6^A-Seq			
	RT-qPCR			
	WB			
	RT-qPCR	METTL3 is targeted by miR-33a attenuating malignant cell proliferation	32 patients and cell lines	Du M 2016 [72]
	WB			
**Brain (Glioblastoma)**	MeRIP/RIP	Knockdown of METLL3/METLL14 and FTO inhibition promotes stem cell renewal and tumorigenesis	Cell lines and animal models	Cui Q 2018 [68]
	m^6^A-Seq			
	m^6^A Dot Blot/Immunobloting			
	IF			
	RT-qPCR			
	m^6^A NorthWestern blot	METTL3 promotes cancer cells maintenance and radioresistance	57 patients, cell lines and animal models	Visvanathan A 2017 [70]
	WB			
	RT-qPCR			
	MeRIP/RIP			
	IHC and IF			
	MeRIP/RIP	ALKBH5 overexpression promotes self-renewal and tumorigenesis through the FOXM1 axis	604 patients, cell lines and animal models	Zhang S 2017 [69]
	m^6^A-Seq			
	WB			
	IHC and IF			
	RT-qPCR			
**Pancreas**	RT-qPCR	METTL3 promotes chemo- and radioresistance	Cell lines	Taketo K 2018 [58]
	WB			
	RT-qPCR	YTHDF2 is upregulated in cancer and regulates EMT	Cell lines	Chen J, 2017 [59]
	IHC			
	WB			
**Biliary tract**	cDNA microarray	WTAP promotes migration and invasion	27 patients, cell lines and animal models	Jo HJ, 2013 [60]
	RT-qPCR			
	WB			
	IHC			
**Stomach**	IHC	FTO overexpression associates with poor prognosis and promotes malignant features	128 patients and cell lines	Xu D 2017 [64]
	RT-qPCR			
	WB			
**Cervix**	m^6^A Dot Blot/Immunobloting	Lower m^6^A levels associate with poor prognosis and malignant features	286 patients, cell lines and animal models	Wang X 2017 [65]
	RT-qPCR			
	WB			
	IHC	FTO overexpression leads to chemo- and radioresistance	30 patients, cell lines and animal models	Zhou S and Bai ZL 2018 [66]
	RT-qPCR			
	WB			
	MeRIP/RIP			
**Endometrium**	m^6^A-seq	METTL14 mutation and METTL3 downregulation leads to decreased m^6^A amount and promotes tumorigenesis by activating AKT signaling	38 patients, cell lines and animal models	Liu J, 2018 [67]
	m^6^A-IP			
	RT-qPCR			
	IHC			
	WB			
**Colorectum**	IHC	YTHDF1 overexpression associates with poor prognosis	63 patients, cell lines and animal models	Nishizawa Y and Kono M 2017 [61]
	RT-qPCR			
	WB			
	IHC	YTHDC2 overexpression promotes metastases by upregulating HIF-1α	72 patients and cell lines	Tanabe A 2016 [62]
	RT-qPCR			
	WB			
**Leukemia**	TCGA database *	Mutations and CNVs in m^6^A-related genes associate with TP53 mutations and poor prognosis in AML patients	191 patients	Kwok CT 2017 [73]
	MeRIP/RIP/ChIP	METTL3 maintains leukemic state	Cell lines and animal models	Barbieri I and Tzelepis K 2017 [75]
	ChIP-seq			
	WB			
	RT-qPCR			
	Flow cytometry			
	m^6^A-seq/RNA-seq	METTL14 promotes leukemogenesis and inhibits hematopoietic stem cell differentiation	Cell lines and animal models	Weng H 2018 [74]
	CLIP			
	ChIP			
	WB			
	RT-qPCR			
	Flow cytometry			
	m^6^A-seq/RNA-seq	FTO promotes leukemogenesis by regulating the ASB2/RARA axis	100 patients, cell lines and animal models	Li Z 2017 [80]
	ChIP			
	WB			
	RT-qPCR			
	m^6^A Dot Blot/Immunobloting			
	Flow cytometry			
	WB	WTAP promotes leukemic cells proliferation and blocks differentiation	511 patients, cell lines and animal models	Bansal H 2014 [77]
	IP			
	RNA-seq			

* In silico analysis only. Abbreviations: cDNA—Complementary DNA; ChIP—Chromatin immunoprecipitation; ChIP-seq—Chromatin immunoprecipitation sequencing; CLIP—Cross-linking and RNA Immunoprecipitation; EMT—Epithelial-to-mesenchymal transition; IF—Immunofluorescence; IHC—Immunohistochemistry; m^6^A-Seq—m^6^A Sequencing; MeRIP—Methylated (m^6^A) RNA Immunoprecipitation; RIP—RNA immunoprecipitation; RNA-seq—RNA sequencing; RT-qPCR—Real-time quantitative Polymerase Chain Reaction; TCGA—The Cancer Genome Atlas; WB—Western blot.

**Table 2:** Most relevant findings of in silico analysis of TCGA database regarding m^6^A-related proteins in urological cancers.

Tumor Model	Sample Size	Most Frequently Deregulated (% of Cases)	Related Alterations (logOR)	Clinicopathological Associations	Survival Impact
**Prostate**	499 tumors	VIRMA (18)	VIRMA + YTHDF3 (co-occurrence, >2)	↑VIRMA and YTHDF3 in stages III/IV (vs. stage II)	No
		YTHDF3 (13)			
	(498 patients)			↑VIRMA and YTHDF3 in GG2-5 (vs. GG1)	
		YTHDC2 (11)			
**Testis**	156 tumors	VIRMA (52)	VIRMA + YTHDF3 (co-occurrence, >3)	↑VIRMA, YTHDF3, METTL4, ALKBH5 and YTHDC1 in SEs (vs. NSTs)	Yes (METTL4, WTAP, YTHDF1)
				↓METTL14 in SEs (vs. NSTs)	
	(150 patients)	YTHDF3 (48)		↑VIRMA, YTHDF3 and METTL4 in stage I (vs. stages II/III)	
**Kidney**	897 tumors	YTHDC2 (21) and RBM15B (14) in ccRCC	VIRMA + YTHDF3 and RBM15B + YTHDC2 (co-occurrence, >3)	↓VIRMA and YTHDC2 in chRCC and pRCC (vs. ccRCC)	Yes (VIRMA, YTHDC2, RBM15B)
		VIRMA (17) and HNRNPA2B1 (17) in chRCC		↑RBM15B in chRCC and pRCC (vs. ccRCC)	
	(895 patients)				
		METTL16 (19), YTHDF1 (19) and RBM15B (14) in pRCC		↑RBM15B and YTHDC2 in stages II–IV (vs. stage I)	
**Bladder**	413 tumors	VIRMA (29)	METTL14 + YTHDC1 and	↑VIRMA, METTL4 and YTHDF3 in High Grade tumors (vs. Low Grade tumors)	Yes (WTAP)
		YTHDF1 (27)			
				↑VIRMA in non-papillary tumors (vs. papillary tumors)	
		METTL4 (21)	METTL3 + HNRNPC (co-occurrence, 2.3 for all)		
	(412 patients)	YTHDF3 (14)			
				↑YTHDC1 in papillary tumors (vs. non-papillary tumors) ↑YTHDC1 in stages I/II (vs. stages III/IV)	
		RBM15 (13)			

Abbreviations: ccRCC—clear cell renal cell carcinoma; chRCC—chromophobe renal cell carcinoma; GG—grade groups; NST—non-seminomatous tumors; OR—odds ratio; pRCC—papillary renal cell carcinoma; SE—seminoma.
